# Optimal esophageal balloon volume for accurate estimation of pleural pressure at end-expiration and end-inspiration: an in vitro bench experiment

**DOI:** 10.1186/s40635-017-0148-z

**Published:** 2017-08-02

**Authors:** Yan-Lin Yang, Xuan He, Xiu-Mei Sun, Han Chen, Zhong-Hua Shi, Ming Xu, Guang-Qiang Chen, Jian-Xin Zhou

**Affiliations:** 1 Department of Critical Care Medicine, Beijing Tiantan Hospital, Capital Medical University, No 6, Tiantan Xili, Dongcheng District, Beijing, 100050 China; 2 Intensive Care Unit, Beijing Electric Power Hospital, Capital Medical University, Beijing, 100073 China; 3 Surgical Intensive Care Unit, Fujian Provincial Clinical College Hospital, Fujian Medical University, Fuzhou, 350001 China

**Keywords:** Esophageal balloon, Balloon volume, Sigmoid regression, Measurement, Bench experiment

## Abstract

**Background:**

Esophageal pressure, used as a surrogate for pleural pressure, is commonly measured by air-filled balloon, and the accuracy of measurement depends on the proper balloon volume. It has been found that larger filling volume is required at higher surrounding pressure. In the present study, we determined the balloon pressure-volume relationship in a bench model simulating the pleural cavity during controlled ventilation. The aim was to confirm whether an optimal balloon volume range existed that could provide accurate measurement at both end-expiration and end-inspiration.

**Methods:**

We investigated three esophageal balloons with different dimensions and materials: Cooper, SmartCath-G, and Microtek catheters. The balloon was introduced into a glass chamber simulating the pleural cavity and volume-controlled ventilation was initiated. The ventilator was set to obtain respective chamber pressures of 5 and 20 cmH_2_O during end-expiratory and end-inspiratory occlusion. Balloon was progressively inflated, and balloon pressure and chamber pressure were measured. Balloon transmural pressure was defined as the difference between balloon and chamber pressure. The balloon pressure-volume curve was fitted by sigmoid regression, and the minimal and maximal balloon volume accurately reflecting the surrounding pressure was estimated using the lower and upper inflection point of the fitted sigmoid curve. Balloon volumes at end-expiratory and end-inspiratory occlusion were explored, and the balloon volume range that provided accurate measurement at both phases was defined as the optimal filling volume.

**Results:**

Sigmoid regression of the balloon pressure-volume curve was justified by the dimensionless variable fitting and residual distribution analysis. All balloon transmural pressures were within ±1.0 cmH_2_O at the minimal and maximal balloon volumes. The minimal and maximal balloon volumes during end-inspiratory occlusion were significantly larger than those during end-expiratory occlusion, except for the minimal volume in Cooper catheter. Mean (±standard deviation) of optimal filling volume both suitable for end-expiratory and end-inspiratory measurement ranged 0.7 ± 0.0 to 1.7 ± 0.2 ml in Cooper, 1.9 ± 0.2 to 3.6 ± 0.3 ml in SmartCath-G, and 2.2 ± 0.2 to 4.6 ± 0.1 ml in Microtek catheter.

**Conclusions:**

In each of the tested balloon, an optimal filling volume range was found that provided accurate measurement during both end-expiratory and end-inspiratory occlusion.

**Electronic supplementary material:**

The online version of this article (doi:10.1186/s40635-017-0148-z) contains supplementary material, which is available to authorized users.

## Background

Esophageal pressure has been used as a surrogate for pleural pressure in the monitoring of respiratory mechanics for many years [1, 2]. Previously, esophageal pressure measurement was mainly used to assess respiratory muscle effort and the work of breathing during spontaneous or assisted ventilation, particularly in patients with difficult weaning [3, 4]. More recently, this technique has been extended to guide lung-protective ventilation in patients with acute respiratory distress syndrome [5–8].

Esophageal pressure is commonly measured by catheter with air-filled balloon, and the accuracy of measurement depends on the proper filling of the balloon [1, 2]. Studies have demonstrated that overfilling of the balloon might overestimate esophageal pressure; in contrast, underfilling might lead to underestimation [9–16]. Theoretically, the optimal balloon volume is the range with minimal discrepancy between the pressures measured inside and outside of the balloon, namely the minimal balloon transmural pressure (*P*_TM_). It has been found that under bench conditions, greater balloon volume is required for pressure transmission at higher surrounding pressure [15]. Therefore, during passive ventilation, the optimal balloon volume obtained during the end-expiratory phase (low pressure) might not be simultaneously suitable for the measurement during the end-inspiratory phase (high pressure), especially in patients with high driving pressure. This possibility warrants further investigation.

Previous studies have shown a nonlinear sigmoid shape of the balloon pressure-volume curve under in vitro and in vivo conditions [9, 11–17]; however, no study has attempted this type of regression analysis. We speculated that the sigmoid fitting of balloon pressure-volume curve might provide an additional method to determine the optimal balloon volume. In the present study, we selected three commercially available esophageal catheters with different balloon dimensions and materials. The balloon pressure and volume data were fitted using a sigmoid regression equation, and the optimal balloon volume was determined under atmospheric pressure and in a dynamic bench model simulating the pleural cavity during volume-controlled ventilation. The overall goodness-of-fit of the sigmoid regression and the accuracy of the balloon volume estimation were assessed. *P*_TM_ was determined, and an acceptable accuracy of measurement was defined as a *P*_TM_ within ±1 cmH_2_O [15]. We aimed to confirm whether an optimal balloon volume range existed that could provide accurate measurement at both end-expiration and end-inspiration.

## Methods

### Esophageal balloon catheters

Three commercially available esophageal balloon catheters with different balloon dimensions and materials were tested: (1) Cooper catheter (LOT 177405, Cooper Surgical, USA) of 5 Fr in diameter and 85 cm in length, enclosed with a polyethylene balloon, and a manufacturer-recommended inflation volume of 1–2 ml; (2) SmartCath-G catheter (LOT 7003300, CareFusion Co., USA) of 16 Fr in diameter and 114 cm in length, with a polyethylene balloon and a manufacturer-recommended inflation volume of 0.5–2.5 ml; and (3) Microtek catheter (LOT 20110815, Microtek Medical B.V., Netherlands) of 8 Fr in diameter and 100 cm in length, with a latex balloon and no manufacturer-recommended inflation volume.

For each type of catheter, six samples were randomly selected. Before the experiment, each balloon was tested for leak by inflating under water. The length and diameter of the balloon were measured in one randomly selected catheter of each type using a Vernier caliper gauge (CD67-S, PM/PS, Mitutoyo Measuring Instruments (Shanghai) Co., Ltd., Shanghai, China). The geometric volume of the balloon was calculated based on the length and diameter of the balloon and using the capsule body as an approximation. Because the feeding tube passes through the inner space of the balloon in the SmartCath-G catheter, this volume was deducted when calculating the balloon geometric volume for this type of catheter.

### Pressure measurements

All pressure measurements were performed using pressure transducers (KT 100D-2, Kleis TEK, Italy, range: ±100 cmH_2_O) connected to an ICU-Lab Pressure Box (ICU Lab, KleisTEK Engineering, Bari, Italy) by 80-cm rigid tube lines. Pressure transducers were calibrated using a water column. Signals were displayed continuously and saved (ICU-Lab 2.5 Software Package, ICU Lab, KleisTEK Engineering, Bari, Italy) on a laptop computer for further analysis, at a sample rate of 200 Hz.

### Balloon volume manipulation at atmospheric pressure

The balloon pressure-volume relationship was determined at atmospheric pressure using a previously reported method with some modifications [12, 13, 16]. The balloon lumen of the catheter was connected with a three-way stopcock for volume manipulation of the balloon, and the other two ways were connected to a 1-ml gas-tight syringe (LOT JM00B25, Runze fluid control equipment, C.O., Ltd., Nanjing, China) and a pressure transducer. The balloon was first inflated up to the geometric volume to unfold the balloon’s wall. The balloon was then deflated by generating a negative pressure of approximately −10 cmH_2_O, and the stopcock was closed. This procedure standardized the residual volume in the balloon, and we arbitrarily defined the balloon volume in this situation as zero inflating volume [12, 13, 16]. Thereafter, the balloon was progressively inflated in 0.5-ml increments. After each volume inflation, balloon pressure was measured after a 3-min equilibration period. Balloon inflation was stopped until the balloon pressure exceeded +10 cmH_2_O. Because the experiment under static conditions was performed at atmospheric pressure, *P*_TM_ was equal to the balloon pressure.

### Balloon volume manipulation under simulated passive ventilation

**Fig. 1:**
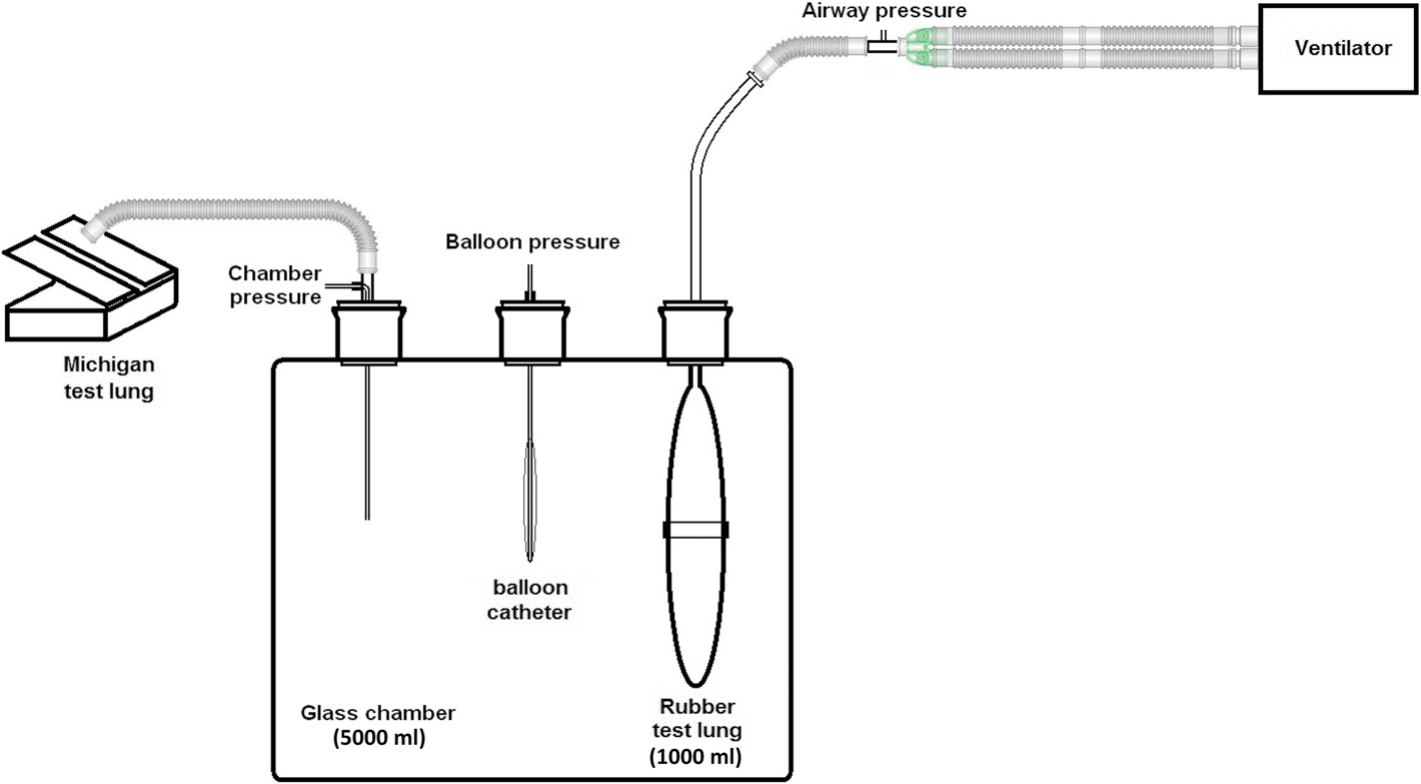
Schematic model with the pleural cavity and the lung

A model was constructed to simulate the pleural cavity and the lung (Fig. 1). The model consisted of a 5000-ml glass chamber (Shuniu Glass Instrument Co., Ltd., Sichuan, China) with three openings, a 1000-ml rubber test lung (Puritan-Bennett Corporation, CA, USA), and a Michigan test lung (Dual Adult Training Test Lung, Model 5600i, Part Number 15090, Michigan Instruments, Grand Rapids, MI, USA). The rubber test lung was sealed in the glass chamber through one opening to simulate the lung and was connected to a Servo-I ventilator (Maquet Critical Care, Solna, Sweden) using an 8.0 ID endotracheal tube (LOT 1892776, Portex Tracheal Tube, Smiths Medical International Ltd., Keene, USA). The glass chamber was connected to the Michigan test lung through the second opening to simulate the pleural cavity with a changeable compliance. The third opening was used to introduce the balloon into the glass chamber and was then sealed. Pressures were measured by different pressure transducers at three positions (Fig. 1): airway pressure at the proximal end of the endotracheal tube, directly measured chamber pressure to represent the surrounding pressure of the balloon, and balloon pressure.

During the bench procedure, mechanical ventilation was set as volume-controlled ventilation with constant flow of 60 l/min; tidal volume of 500 ml; inspiratory time of 0.5 s with inspiratory pause time of 0.5 s; respiratory rate of 20 breaths/min; and positive end-expiratory pressure (PEEP) of 5 cmH_2_O. The inspiratory trigger was set at −20 cmH_2_O to avoid auto-triggering. The compliance of the Michigan test lung was set to 20 ml/cmH_2_O. These ventilator and test lung settings produced a plateau airway pressure of approximately 40 cmH_2_O and a chamber pressure of 20 cmH_2_O.

After the balloon was randomly introduced into the glass chamber described above, the model was tested by two methods to confirm that there was no systematic leak: (1) expiratory tidal volume no less than inspiratory tidal volume during mechanical ventilation and (2) decrease in airway pressure of no less than 1 cmH_2_O during 30 s of end-expiratory occlusion at 30 cmH_2_O of PEEP.

A three-way stopcock was connected to the balloon lumen of the catheter. Mechanical ventilation was not terminated during balloon manipulation to mimic an actual clinical scenario. The balloon was first deflated by generating a negative pressure, followed by opening the balloon to the atmosphere for 3 min to standardize the residual volume remaining in the balloon and the catheter lumen. The balloon was then progressively inflated in 0.5-ml increments to a volume that resulted in a deviation in the balloon pressure from the chamber pressure that exceeded 1 cmH_2_O. Balloon inflation was performed at end-expiration. At each tested balloon volume, after 3-min equilibration period, end-expiratory and end-inspiratory occlusion was performed (each for 10 s), and balloon pressure and chamber pressure were simultaneously measured during the last second.

### Balloon volume determination by sigmoid regression

Balloon volume was plotted against balloon pressure, and these two variables were fitted to the following sigmoid regression equation [18–20]:

$$ \mathrm{Balloon}\ \mathrm{volume}=a+\frac{b}{1+{e}^{-\left(\mathrm{Balloon}\ \mathrm{pressure}-c\right)/d}} $$ (1)

**Fig. 2:**
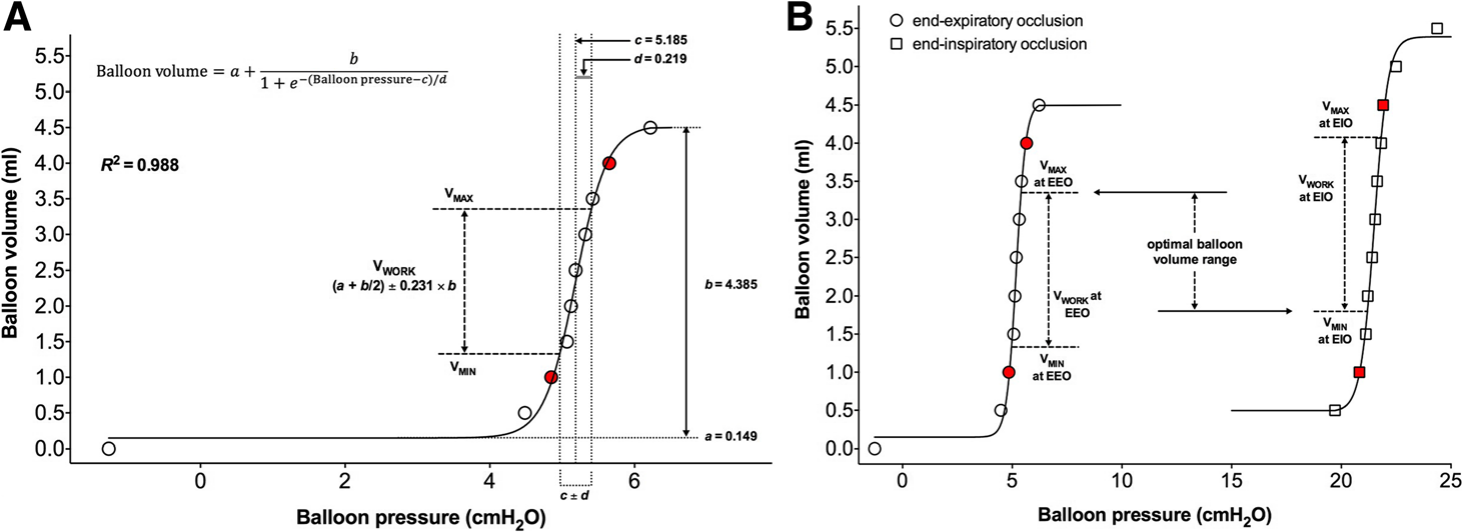
An example of the fitted balloon volume-pressure curve using the sigmoid regression equation. **a** An example of the fitting of balloon pressure and balloon volume in a SmartCath-G balloon at end-expiratory occlusion during simulated mechanical ventilation. *Circles* are individual data points. The *solid line* represents the fitted curve. The equation (shown on the *top* of the figure) has four fitting parameters: *a*, in units of volume (ml), representing the lower asymptote of the fitted sigmoid curve; *b*, also in units of volume (ml), representing the vertical distance from the lower to the upper asymptote; *c* (cmH_2_O), representing the pressure at the midpoint of the sigmoid curve where the concavity changes direction; and *d* (cmH_2_O), is proportional to the minimal balloon pressure change as balloon volume increasing. We defined the balloon volume at balloon pressure of *c* − *d* as the minimal inflating volume (*V*_MIN_, equal to [*a* + *b*/2] − 0.231*b*) and *c* + *d* as the maximal volume (*V*_MAX_, equal to [*a* + *b*/2] + 0.231*b*). Within these two points, the balloon pressure change was minimal during the inflation of the balloon. Balloon working volume (*V*_WORK_) was defined as the difference between *V*_MIN_ and *V*_MAX_. **b** The analysis of *V*_WORK_s during end-expiratory (EEO) and end-inspiratory occlusion (EIO) in the same balloon in **a**. The *V*_MIN_ and *V*_MAX_ both satisfying the two phases were defined as the low and the high limit of the optimal balloon volume range

The balloon pressure-volume curve was fitted using the Levenberg-Marquardt iterative algorithm, which was set to run until the change in the sum of squared residuals was lower than 10^−8^. An example scatter graph with fitted line is shown in Fig. 2a. Equation 1 has four fitting parameters: *a*, in units of volume (ml), representing the lower asymptote of the fitted sigmoid curve; *b*, also in units of volume (ml), representing the vertical distance from the lower to the upper asymptote; *c* (cmH_2_O), representing the pressure at the midpoint of the sigmoid curve where the concavity changes direction; and *d* (cmH_2_O), which is proportional to the minimal balloon pressure change as the balloon volume increases. According to the characteristic features of the sigmoid curve, the maximal upward and downward curvature changes occurred at the lower and upper inflection points with balloon pressures equal to *c* ± 1.317*d* (Fig. 2a) [21]. Within these two inflection points, the balloon pressure change was minimal during the progressively inflation of the balloon. We selected a narrower range for the estimation of optimal balloon volume and defined the balloon volume at balloon pressure of *c* − *d* as the minimal inflating volume (*V*_MIN_, equal to [*a* + *b*/2] − 0.231*b*) and *c* + *d* as the maximal volume (*V*_MAX_, equal to [*a* + *b*/2] + 0.231*b*). The balloon *P*_TM_ values at *V*_MIN_ and *V*_MAX_ were calculated as the estimated balloon pressure minus the averaged chamber pressure. Acceptable accuracy of measurement was defined as a *P*_TM_ within ±1 cmH_2_O [15]. Balloon working volume (*V*_WORK_) was calculated as the difference between *V*_MIN_ and *V*_MAX_. Under atmospheric condition, balloon elastance was estimated as the difference in balloon pressure between *V*_MIN_ and *V*_MAX_ divided by *V*_WORK_.

In each tested balloon, *V*_WORK_ values during end-expiratory and end-inspiratory occlusion were explored, and the *V*_MIN_ and *V*_MAX_ providing accurate measurement at both phases were defined as the low and the high limits of the optimal balloon volume range (Fig. 2b). Visual inspection of the intermediate linear section of the balloon pressure-volume curve was also performed using the method reported by Mojoli et al. [17]. Balloon *P*_TM_ values at *V*_MIN_ and *V*_MAX_ obtained using the visual inspection method were also calculated.

### Statistical analysis

In the balloon pressure-volume curve analysis, the best-fit coefficient *R* ^2^ and the bias-corrected Akaike information criterion (AICc) of the sigmoid regression were calculated [22]. To assess the overall goodness-of-fit in the regression equation of each type of balloon using each corresponding fitted equation, the balloon volume and balloon pressure were transformed to dimensionless variables (balloon volume − *a*)/*b* and (balloon pressure − *c*)/*d*, which allowed these variables to be plotted on the same horizontal and vertical axes. These two dimensionless variables were fitted to the following equation [19, 20]:

$$ \left(\mathrm{balloon}\ \mathrm{volume}-a\right)/b=\frac{1}{1+{e}^{-\left(\mathrm{balloon}\ \mathrm{pressure}-c\right)/d}} $$ (2)

The mean and standard deviation (SD) of the residual for (balloon volume − *a*)/*b* were calculated and plotted against (balloon pressure − *c*)/*d*.

Categorical variables were expressed as counts and percentages. Continuous data were checked for normal distribution using the Kolmogorov-Smirnov test and were presented as mean ± SD or as median (25th to 75th percentile) as appropriate. Balloon volume data at atmospheric pressure were compared across different balloon types using one-way ANOVA and the Student-Newman-Keuls test for pairwise comparisons. Under dynamic conditions during simulated passive ventilation, one-way ANOVA was also used to compare chamber pressure and balloon pressure at different balloon volume. Repeated measures of analysis of variance was used to compare balloon volume data across different balloon types and between end-expiratory and end-inspiratory occlusion. The paired *t* test was used to compare the difference in the absolute *P*_TM_ at *V*_MIN_ or *V*_MAX_ between the sigmoid fitting and visual inspecting methods.

All tests of significance were at the 5% significance level. Analyses were conducted using SPSS V.20.0 (SPSS Inc., Chicago, IL, USA).

## Results

Sigmoid fitting parameters for all tested balloons and under all bench conditions are shown in Additional file 1: Table S1. When data obtained under all tested conditions for each type of balloon were pooled and normalized according to the parameters in each corresponding regression equation (Eq. 1), the dimensionless variable (balloon volume − *a*)/*b* was fitted closely with (balloon pressure − *c*)/*d* along the standard sigmoid curve (Additional file 1: Figure S1). The mean ± SD of residual of (balloon volume − *a*)/*b* was 0.000047 ± 0.0358, 0.00000 ± 0.0554, and 0.000028 ± 0.0648 for the Cooper, SmartCath-G, and Microtek catheters, respectively. Residuals of (balloon volume − *a*)/*b* were scattered evenly against (balloon pressure − *c*)/*d* for the Cooper catheter, but a statistically positive correlation was found for the SmartCath-G and the Microtek catheters (Additional file 1: Figure S2).

**Fig. 3:**
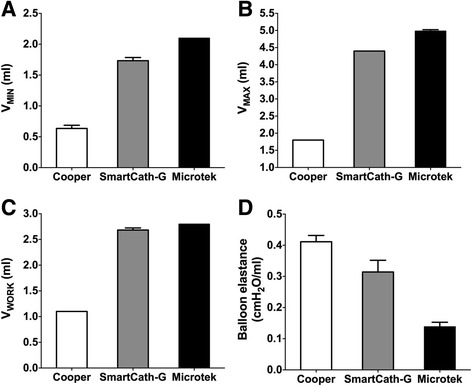
**a**–**d** Balloon volume data and balloon elastance at atmosphere. Data are shown as mean and standard deviation. Significant difference existed in minimal (*V*_MIN_, **a**), maximal balloon inflating volume (*V*_MAX_, **b**), working volume (*V*_WORK_, **c**), and balloon elastance across different balloons (*p* < 0.001, **d**). All *p* values are <0.001 in pairwise comparison

Table 1 shows the characteristics of the tested balloons. The geometric volume of the balloon was 2.8 ml for the Cooper, 5.3 ml for the SmartCath-G, and 7.5 ml for the Microtek catheter. At atmospheric pressure, there were significant differences in *V*_MIN_, *V*_MAX_, *V*_WORK_, and balloon elastance across the different balloon types (*p* < 0.001, Fig. 3).

**Table 1:** Balloon material and dimension characteristics

	Cooper	SmartCath-G	Microtek
Balloon material	Polyethylene	Polyethylene	Latex
Length of the balloon (mm)	92.5	109.3	105.2
Diameter of the balloon (mm)	6.3	10.4	9.7
Geometric volume of the balloon^a^ (ml)	2.8	5.3	7.5
Recommended inflating volume (ml)	1.0–2.0	0.5–2.5	Not available

^a^Geometric volume of the balloon was calculated by the length and diameter of the balloon, using the capsule body as approximation. Because the feeding tube passed through the inner space of the balloon in SmartCath-G catheter, this volume was deducted in the calculation of balloon geometric volume in this type of catheter

**Fig. 4:**
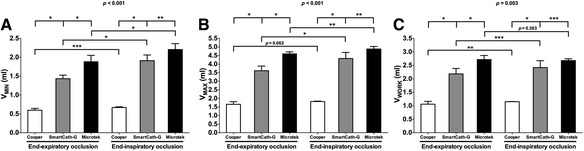
Balloon volume data during simulated mechanical ventilation. Data are shown as mean and standard deviation. There were significant differences in minimal (**a**; *V*_MIN_, *p* < 0.001) and maximal (**b**; *V*_MAX_, *p* < 0.001) balloon inflating volume and the working volume (**c**; *V*_WORK_, *p* = 0.003) across different balloon types and between end-expiratory and end-inspiratory occlusion. Results of pairwise comparison are shown: *p < 0.001, **p < 0.01, and ***p < 0.05

**Fig. 5:**
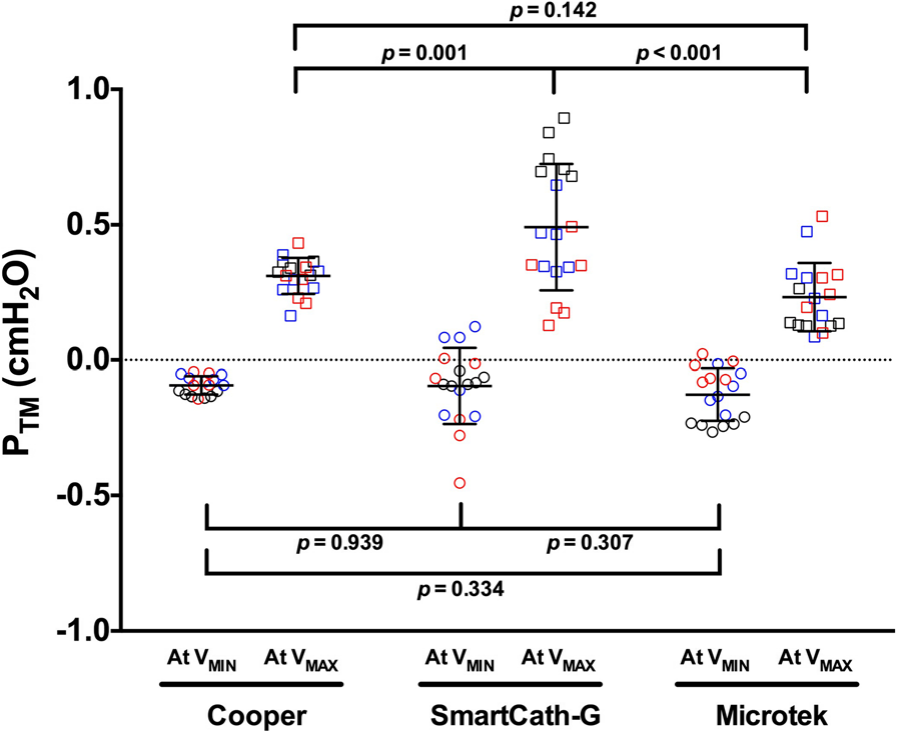
Balloon transmural pressure under all bench conditions. Balloon transmural pressure (*P*_TM_) at minimal (*V*_MIN_) and maximal balloon inflating volume (*V*_MAX_) under atmosphere (*black*) and end-expiratory (*blue*) and end-inspiratory occlusion (*red*) during simulated passive ventilation. All *P*_TM_ were within ±1 cmH_2_O. Mean and standard deviation are also shown

There was no systematic leak in the dynamic model during simulated passive ventilation. With progressive increments of balloon injection volume, the chamber pressure remained stable, but balloon pressure increased significantly in each type of catheter at end-expiratory or end-inspiratory occlusion (all *p* < 0.001) (Additional file 2: Figure S3). Balloon volume data during dynamic ventilation are shown in Fig. 4. There were significant differences in *V*_MIN_, *V*_MAX_, and *V*_WORK_ across different balloon types and between end-expiratory and end-inspiratory occlusion (*p* = 0.003 to <0.001). During either end-expiratory or end-inspiratory occlusion, *V*_MIN_, *V*_MAX_, and *V*_WORK_ were in a significant ascending order of Cooper < SmartCath-G < Microtek catheters, in accordance with the sequence of geometric volume. The three balloon volume parameters during end-inspiratory occlusion were significantly larger than those during end-expiratory occlusion, except for *V*_MAX_ in the Cooper (*p* = 0.062) and *V*_WORK_ in the Microtek (*p* = 0.363) (Fig. 4). In all tested balloons, all *P*_TM_ values under the different tested conditions (atmospheric pressure and simulated ventilation) were within ±1.0 cmH_2_O (Fig. 5). All *V*_MIN_ and *V*_MAX_ determined by the sigmoid regression were within the intermediate linear section obtained by visually inspecting the balloon pressure-volume curve (Additional file 3: Table S2). For either *V*_MIN_ or *V*_MAX_, absolute *P*_TM_ values as measured by the sigmoid fitting were lower than those measured by the intermediate linear section inspection (Additional file 3: Figure S4).

**Fig. 6:**
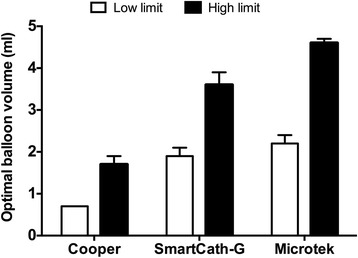
The low and high limit of optimal balloon volumes that provide accurate measurement during both end-expiratory and end-inspiratory occlusion. The low and high limits were defined as the minimal and maximal balloon inflating volume that provided accurate measurement during both end-expiratory and end-inspiratory occlusion

For each tested balloon, an optimal balloon volume range existed that provided accurate measurement during both end-expiratory and end-inspiratory occlusion, which was 0.7 ± 0.0 to 1.7 ± 0.2 ml for the Cooper, 1.9 ± 0.2 to 3.6 ± 0.3 ml for the SmartCath-G, and 2.2 ± 0.2 to 4.6 ± 0.1 ml for the Microtek catheter (Fig. 6).

## Discussion

In the present study, we used a mathematical method, sigmoid regression, to analyze the balloon pressure-volume curve and to determine the optimal balloon volume under different bench conditions. Our main findings are:

(1) An optimal balloon volume range exists that provides accurate measurement at both end-expiration and end-inspiration for each of the tested balloon.

(2) The sigmoid regression fits the balloon pressure-volume curve excellently and enables an accurate estimation of the optimal balloon volume. This method provides an additional way to determine the optimal balloon volume.

Proper titration of balloon volume is critical for the accurate measurement of esophageal pressure [1, 2]. In bench studies, the optimal balloon volume was defined as a volume range in which *P*_TM_ was within a certain threshold [14, 15]. In the present study, we estimated the optimal balloon volume and range using a sigmoid fitting of balloon pressure-volume curve, which has been used to predict the lower and upper inflection points in the respiratory system pressure-volume curve analysis [19, 20]. After normalization based on parameters of the regression equation, the dimensionless variables of balloon pressure and balloon volume were closely distributed along the standard sigmoid curve, and the mean residual for the dimensionless volume variables was minor (Additional file 1: Figure S1 and S2). More importantly, all measured *P*_TM_ values at *V*_MIN_ and *V*_MAX_ were within ±1 cmH_2_O (Fig. 5), which has been used as the threshold in previous bench study [15]. Together, these results indicate that the sigmoid regression of balloon pressure and volume can be used to accurately estimate the optimal filling volume.

In the present study, in addition to testing the balloons under atmospheric condition, we also analyzed the balloon pressure-volume relationship during simulated passive ventilation in a respiratory system model (Fig. 1), which has been reported for educational purposes [23, 24]. We employed this model in an attempt to mimic actual clinical scenarios. Using our sigmoid fitting method, we confirmed previous two major findings regarding the relationship between balloon filling volume and geometric volume and surrounding pressure [14, 15]. First, the balloon filling volume is determined by the geometric volume of the balloon (Fig. 3): the larger the geometric volume, the larger the balloon filling volume that is needed to reflect surrounding pressure. Second, the balloon filling volume required for accurate measurement increases as the balloon surrounding pressure increases (Fig. 4). The latter finding is of clinical significance during esophageal monitoring in passive patients under controlled ventilation. Because pleural pressure increases during inspiration, the balloon volume suitable for measuring during the end-expiratory phase may not be adequate for measurement during the end-inspiratory phase. Fortunately, this did not occur in our bench model with simulated pleural pressures ranging from 5 cmH_2_O during end-expiratory occlusion to 20 cmH_2_O during end-inspiratory occlusion. An optimal volume range existed that enabled accurate measurement at both phases (Fig. 6).

The slope of the intermediate section is minimal under in vitro conditions and is related to balloon elastance [15, 16], but is usually inclined in vivo due to esophageal elastance [17]. We estimated the balloon elastance based on the fitted balloon pressure and volume data, and our results (0.1 to 0.4 cmH_2_O/ml, Fig. 3d) were comparable to those reported by Cross et al. [16]. Further investigation is warranted to determine whether this method can improve the accuracy of esophageal elastance measurement and esophageal pressure calibration at the bedside.

In clinical settings, because it is difficult to obtain the *P*_TM_, the proper balloon volume is usually determined by visual inspecting the intermediate linear section of the balloon pressure-volume curve [17]. Compared to the visual inspection method, fitting the data to a sigmoid curve yielded *V*_WORK_ with a narrower range and *P*_TM_ closer to zero (Additional file 3: Table S2 and Figure S4). These results suggested that our introduced method might provide a stricter estimation of the optimal balloon volume. Additionally, sigmoid fitting is objective and based on mathematics. However, because the sigmoid fitting method was only investigated in a bench model in the present study, further study is needed to determine whether this method can improve the assessment of optimal balloon volume. Since a relatively low adequate balloon volume is recommended to avoid overfilling [2, 11], we suggest using the low limit of the optimal balloon volume range (Fig. 6), thus avoiding overfilling of the balloon during expiration and simultaneously avoiding underfilling during inspiration.

There are limitations to the present study. First, in our bench model, we only simulated the pleural cavity without the esophagus. It has been shown that esophageal wall elastance significantly influences esophageal pressure monitoring [17]. Therefore, our findings cannot be directly translated into clinical practice. Moreover, the procedure was only performed during controlled ventilation, and the findings cannot be applied to patients with spontaneous breathing. Second, the high balloon surrounding pressure simulated in the present study was somewhat lower than the end-inspiratory esophageal pressure in patients with acute respiratory distress syndrome during passive ventilation [5–7]. The titration of balloon volume needs further investigation in clinical studies, especially in patients with relatively high esophageal pressure and airway driving pressure.

## Conclusions

The balloon pressure-volume curve is fitted well by sigmoid regression and provides an accurate estimation of the balloon volume. Careful titration of the filling volume should be performed in clinical practice because the adequate balloon volume is discrepant among different types of balloon at different surrounding pressures. In each of the tested balloon, an optimal filling volume range exists that provides accurate measurement during both end-expiratory and end-inspiratory occlusion.

## Additional files

Additional file 1: Table S1. Sigmoid fitting parameters at atmospheric pressure and end-expiratory and end-inspiratory occlusion. **Figure S1.** Fitting of the dimensionless variables in balloon pressure-volume sigmoid regression equation. **Figure S2.** Plot of residual of (balloon volume − *a*)/*b* predicted by a standard sigmoid fitting against (balloon pressure − *c*)/*d*. (PDF 599 kb)

Additional file 2: Figure S3. Chamber pressure and balloon pressure during balloon volume manipulation at atmospheric pressure and under simulated passive ventilation. (PDF 212 kb)

Additional file 3: Table S2. Minimal and maximal balloon inflating volume estimated by sigmoid fitting and by visual inspection of the intermediate linear section of the balloon pressure-volume curve during end-expiratory and end-inspiratory occlusion. **Figure S4.** Absolute balloon transmural pressure at minimal and maximal balloon inflating volume. (PDF 329 kb)

## Abbreviations

PEEP: Positive end-expiratory pressure
*P*_TM_: Balloon transmural pressure
SD: Standard deviation
*V*_MAX_: Maximal balloon inflating volume
*V*_MIN_: Minimal balloon inflating volume
*V*_WORK_: Balloon working volume
