# Supplementary material for: Optimal esophageal balloon volume for accurate estimation of pleural pressure at end-expiration and end-inspiration: an in vitro bench experiment
Source: Intensive Care Med Exp. 2017 Aug 2;5:35. doi: 10.1186/s40635-017-0148-z (PMC5540740; doi:10.1186/s40635-017-0148-z)

Optimal esophageal balloon volume for accurate estimation of pleural pressure at end-expiration and end-inspiration: an *in vitro* bench experiment

Yan-Lin Yang, Xuan He, Xiu-Mei Sun, Han Chen, Zhong-Hua Shi, Ming Xu, Guang-Qiang Chen, Jian-Xin Zhou

Additional file 1

Table S1. Sigmoid fitting parameters at atmospheric pressure (ATM), end-expiratory occlusion (EEO) and end-inspiratory occlusion (EIO).

Cooper

ATM	*a*	*b*	*c*	*d*	R^2	AICc
No1	-0.016	2.485	0.119	0.244	0.994	32.2
No2	-0.022	2.480	0.125	0.240	0.993	33.0
No3	-0.016	2.486	0.114	0.249	0.995	31.4
No4	-0.026	2.475	0.107	0.219	0.992	34.0
No5	-0.024	2.475	0.100	0.240	0.991	34.6
No6	-0.021	2.482	0.090	0.223	0.994	32.5
EEO	*a*	*b*	*c*	*d*	R^2	AICc
No1	-0.036	2.393	5.483	0.220	0.973	41.4
No2	-0.035	2.394	5.340	0.210	0.976	40.6
No3	-0.016	2.488	5.213	0.175	0.997	29.0
No4	-0.029	2.471	5.211	0.173	0.990	35.3
No5	0.002	2.004	5.168	0.166	1.000	-
No6	-0.003	2.000	5.143	0.114	1.000	-
EIO	*a*	*b*	*c*	*d*	R^2	AICc
No1	-0.008	2.501	22.208	0.210	0.999	18.0
No2	0.009	2.492	21.958	0.196	0.985	37.9
No3	0.012	2.492	21.907	0.136	0.990	35.6
No4	0.006	2.503	21.828	0.130	0.968	42.4
No5	-0.005	2.489	22.047	0.262	0.994	32.7
No6	-0.029	2.466	21.872	0.227	0.981	39.2

SmartCath-G

ATM	*a*	*b*	*c*	*d*	R^2	AICc
No1	0.185	5.762	0.373	0.468	0.983	14.4
No2	0.156	5.789	0.402	0.491	0.983	14.5
No3	0.192	5.728	0.328	0.416	0.979	17.9
No4	0.193	5.779	0.307	0.390	0.985	13.1
No5	0.250	5.745	0.333	0.373	0.985	12.9
No6	0.223	5.750	0.308	0.371	0.982	15.2
EEO	*a*	*b*	*c*	*d*	R^2	AICc
No1	0.095	5.527	5.782	0.425	0.988	8.3
No2	0.269	4.362	5.611	0.194	0.989	6.3
No3	0.179	4.582	5.346	0.130	0.981	12.5
No4	0.261	4.566	5.340	0.112	0.973	16.4
No5	0.149	4.385	5.185	0.219	0.992	3.2
No6	0.028	4.897	5.337	0.336	0.987	8.3
EIO	*a*	*b*	*c*	*d*	R^2	AICc
No1	0.564	5.944	22.512	0.286	0.985	11.4
No2	0.717	4.374	21.893	0.094	0.976	11.8
No3	0.405	5.160	21.682	0.097	0.888	34.6
No4	0.578	5.343	21.589	0.094	0.874	36.0
No5	0.482	4.909	21.505	0.314	0.995	-3.8
No6	0.279	5.654	21.859	0.473	0.982	12.5

Microtek

ATM	*a*	*b*	*c*	*d*	R^2	AICc
No1	0.526	6.032	-0.070	0.195	0.983	14.1
No2	0.499	6.133	-0.047	0.186	0.984	13.9
No3	0.504	6.100	-0.059	0.185	0.984	13.8
No4	0.502	6.102	-0.055	0.184	0.983	14.5
No5	0.472	6.149	0.027	0.237	0.984	14.0
No6	0.498	6.134	-0.050	0.185	0.984	13.4
EEO	*a*	*b*	*c*	*d*	R^2	AICc
No1	0.495	5.660	5.538	0.339	0.988	13.2
No2	0.421	5.935	5.926	0.159	0.965	24.9
No3	0.135	6.221	5.402	0.181	0.897	40.0
No4	0.601	5.405	5.303	0.207	0.989	14.4
No5	-0.001	6.211	5.341	0.156	0.966	24.4
No6	0.166	5.874	5.211	0.068	0.967	23.9
EIO	*a*	*b*	*c*	*d*	R^2	AICc
No1	0.780	5.824	22.299	0.299	0.948	27.7
No2	0.838	5.639	22.862	0.141	0.953	26.4
No3	0.606	5.919	22.440	0.157	0.833	42.9
No4	0.769	5.700	22.171	0.167	0.970	20.7
No5	0.417	5.993	22.188	0.138	0.973	19.5
No6	0.479	5.672	21.743	0.052	0.940	29.6

AICc represents the bias-corrected Akaike Information Criterion of the sigmoid regression

Figure S1.

Fitting of the dimensionless variables in balloon pressure-volume sigmoid regression equation.

To assess the overall goodness-of-fit in the regression equation of each type of balloon using each corresponding fitted equation, the balloon volume and balloon pressure were transformed to dimensionless variables (balloon volume − a)/b and (balloon pressure − c)/d, which allowed to be plotted on the same horizontal and vertical axes. These two dimensionless variables were fitted by the equation: $(\text{balloon volume} - a)/b = \frac{1}{1+e^{-(\text{balloon pressure} - c)/d}}$

A: Cooper, B: SmartCath-G, C: Microtek. Data under atmosphere (ATM), end-expiratory (EEO) and end-inspiratory occlusion (EIO) during simulated passive ventilation are pooled together. The solid line represents the standard sigmoid curve fitted by the equation shown on the top of the figure.

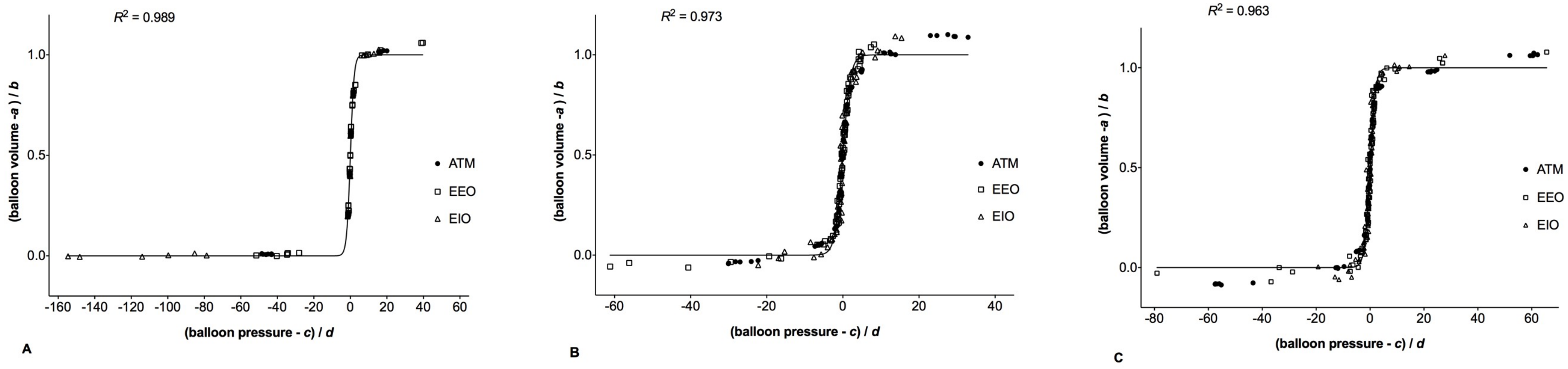

Figure S2.

Plot of residual of (balloon volume - *a*)/*b* predicted by a standard sigmoid fitting against (balloon pressure - *c*)/*d*.

The mean and standard deviation (SD) of the residual in (balloon volume − *a*)/*b* were calculated and plotted against (balloon pressure − *c*)/*d*. The mean (solid line) and standard deviation (SD) of the residual, and the correlation (dotted line) between the two variables are shown.

A: Cooper catheter, B: SmartCath-G catheter, C: Microtek.

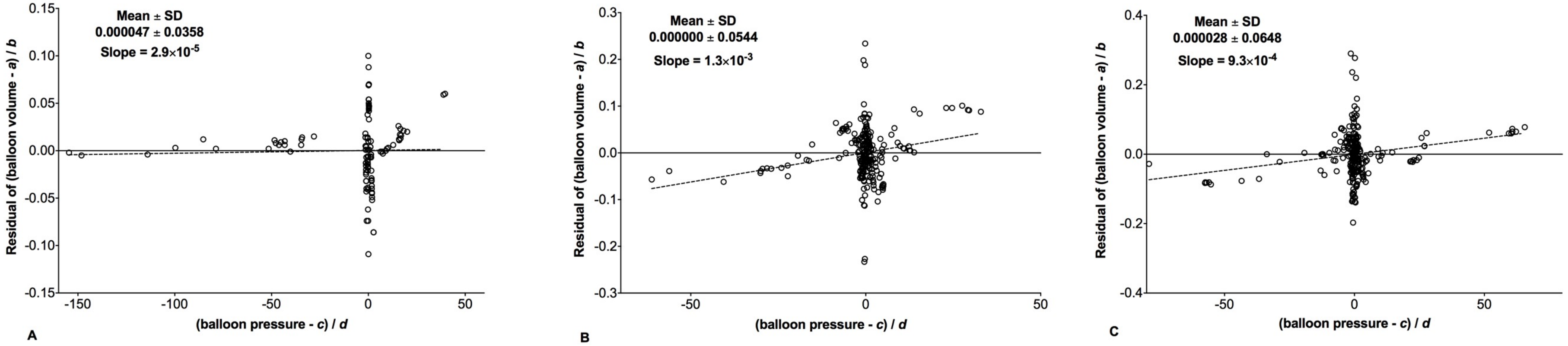

Supplement: Supplementary file 1 — Sigmoid fitting parameters at atmospheric pressure and end-expiratory and end-inspiratory occlusion. Figure S1. Fitting of the dimensionless variables in balloon pressure-volume sigmoid regression equation. Figure S2. Plot of residual of (balloon volume − a)/b predicted by a standard sigmoid fitting against (balloon pressure − c)/d. (PDF 599 kb) [file 40635_2017_148_MOESM1_ESM.pdf]
