# Supplementary material for: Optimal esophageal balloon volume for accurate estimation of pleural pressure at end-expiration and end-inspiration: an in vitro bench experiment
Source: Intensive Care Med Exp. 2017 Aug 2;5:35. doi: 10.1186/s40635-017-0148-z (PMC5540740; doi:10.1186/s40635-017-0148-z)

Optimal esophageal balloon volume for accurate estimation of pleural pressure at end-expiration and end-inspiration: an *in vitro* bench experiment

Yan-Lin Yang, Xuan He, Xiu-Mei Sun, Han Chen, Zhong-Hua Shi, Ming Xu, Guang-Qiang Chen, Jian-Xin Zhou

Additional file 2

Figure S3.

Chamber pressure and balloon pressure during balloon volume manipulation under simulated passive ventilation.

Data are shown as mean and standard deviation. The chamber pressure (P_C) and balloon pressure (P_B) during balloon volume manipulations at atmospheric pressure (ATM) and under simulated passive ventilation at end-expiratory (EEO) and end-inspiratory (EIO) occlusion are shown. A: Cooper; B: SmartCath-G; C: Microtek.

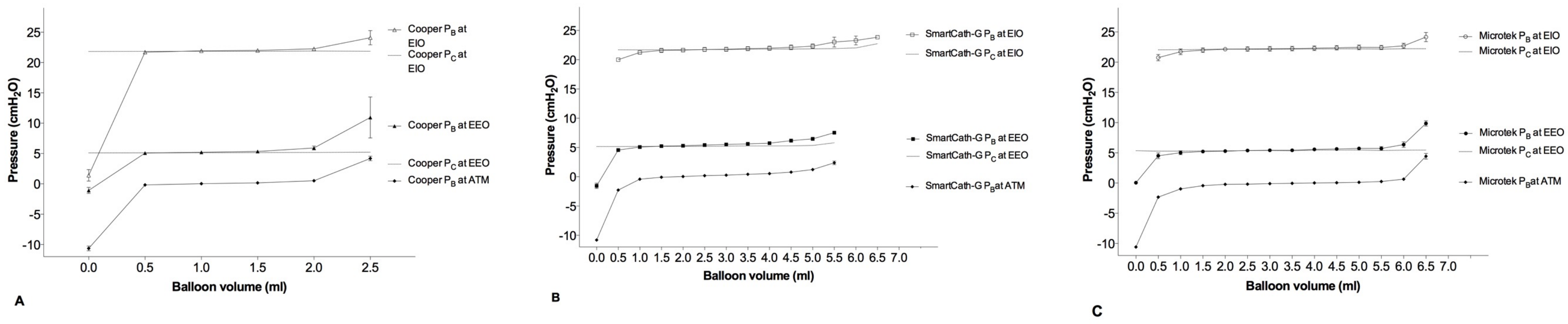

Supplement: Supplementary file 2 — Chamber pressure and balloon pressure during balloon volume manipulation at atmospheric pressure and under simulated passive ventilation. (PDF 212 kb) [file 40635_2017_148_MOESM2_ESM.pdf]
