# Supplementary material for: Optimal esophageal balloon volume for accurate estimation of pleural pressure at end-expiration and end-inspiration: an in vitro bench experiment
Source: Intensive Care Med Exp. 2017 Aug 2;5:35. doi: 10.1186/s40635-017-0148-z (PMC5540740; doi:10.1186/s40635-017-0148-z)

Optimal esophageal balloon volume for accurate estimation of pleural pressure at end-expiration and end-inspiration: an *in vitro* bench experiment

Yan-Lin Yang, Xuan He, Xiu-Mei Sun, Han Chen, Zhong-Hua Shi, Ming Xu, Guang-Qiang Chen, Jian-Xin Zhou

Additional file 3

Table S2. Minimal and maximal balloon volume (V_{MIN} and V_{MAX}) estimated by sigmoid fitting and by visual inspecting of intermediate linear section of the balloon pressure-volume curve during end-expiratory (EEO) and end-inspiratory occlusion (EIO)

		EEO				EIO			
		By sigmoid fitting		By visual inspection		By sigmoid fitting		By visual inspection	
		V_{MIN}	V_{MAX}	V_{MIN}	V_{MAX}	V_{MIN}	V_{MAX}	V_{MIN}	V_{MAX}
Cooper	No1	0.6	1.7	0.5	2.0	0.7	1.8	0.5	2.0
	No2	0.6	1.7	0.5	2.0	0.7	1.8	0.5	2.0
	No3	0.7	1.8	0.5	2.0	0.7	1.8	0.5	2.0
	No4	0.6	1.8	0.5	2.0	0.7	1.8	0.5	2.0
	No5	0.5	1.5	0.5	1.5	0.7	1.8	0.5	2.0
	No6	0.5	1.5	0.5	1.5	0.6	1.8	0.5	2.0
SmartCath-G	No1	1.6	4.1	1.0	5.0	2.2	4.9	1.5	5.0
	No2	1.4	3.5	1.0	4.0	1.9	3.9	1.5	5.0
	No3	1.4	3.5	1.0	4.0	1.8	4.2	1.0	4.0
	No4	1.5	3.6	1.0	4.0	2.0	4.5	1.5	5.5
	No5	1.3	3.4	1.0	4.0	1.8	4.1	1.0	4.5
	No6	1.3	3.6	0.5	4.0	1.8	4.4	1.0	5.0
Microtek	No1	2.0	4.6	1.5	5.5	2.3	5.0	2.0	5.5
	No2	2.0	4.8	1.0	5.5	2.4	5.0	2.5	5.5
	No3	1.8	4.7	1.5	5.5	2.2	4.9	1.5	5.0
	No4	2.1	4.6	1.5	5.5	2.3	4.9	2.0	6.0
	No5	1.7	4.5	1.0	5.5	2.0	4.8	1.5	5.0
	No6	1.7	4.5	1.0	5.5	2.0	4.6	1.5	5.0

All V_{MIN} and V_{MAX} determined by the sigmoid fitting were within the intermediate linear section obtained by visually inspecting of the balloon pressure-volume curve.

Figure S4. Absolute balloon transmural pressure (P_{TM}) at minimal (V_{MIN}) and maximal balloon inflating volume (V_{MAX})

Absolute P_{TM}s determined by sigmoid fitting was significantly lower than those by intermediate linear section inspecting. Mean and standard deviation are also shown.

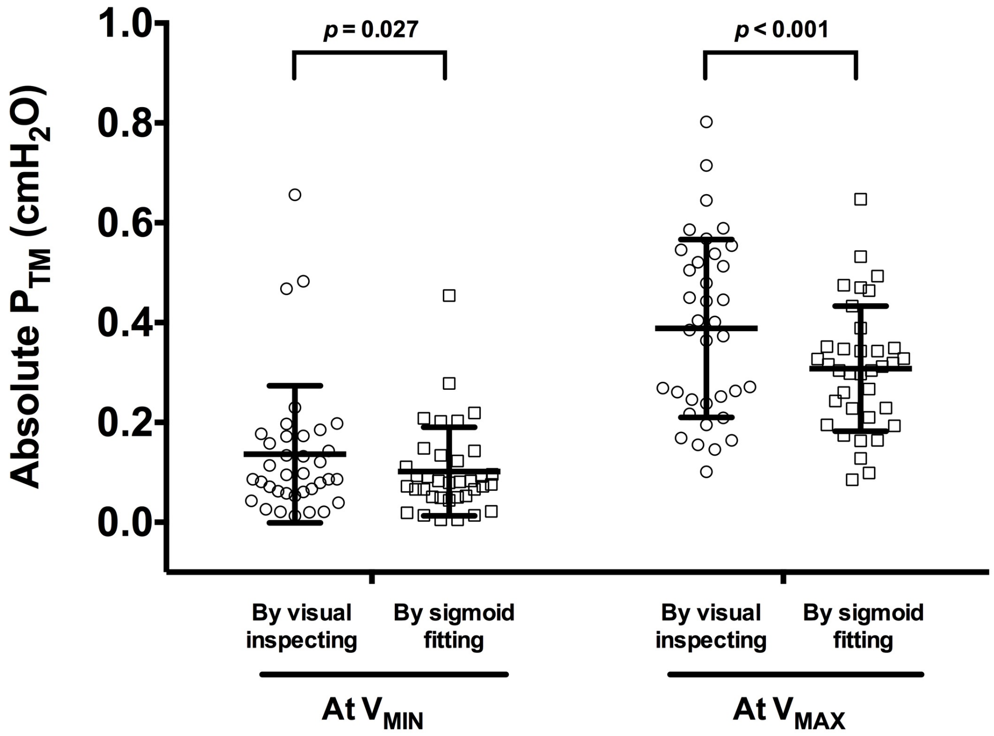

Supplement: Supplementary file 3 — Minimal and maximal balloon inflating volume estimated by sigmoid fitting and by visual inspection of the intermediate linear section of the balloon pressure-volume curve during end-expiratory and end-inspiratory occlusion. Figure S4. Absolute balloon transmural pressure at minimal and maximal balloon inflating volume. (PDF 329 kb) [file 40635_2017_148_MOESM3_ESM.pdf]
